# Explanation

**Published:** 1856-04

**Authors:** 


*Explanation.*—We insert the following with pleasure as explanatory of a good-natured controversy in our pages.—ED.

A correspondent of the *Buffalo Medical Journal* takes us to task (for we alone bear the responsibility), for publishing the portrait and biography of Dr. Davis, which appeared in the REPORTER for last May. We hesitate not to leave it to the judgment of "R." himself, if the history of the American Medical Association would have been complete on our plan *without* a portrait and biography of its originator. It is a significant fact, that the communication of "R." dates from Detroit, and but for the assurance of the editor of the *Buffalo Journal* that its writer "has no acquaintance with, or prejudice against" Dr. Davis, we should have laid it at the door of a certain friend of the Medical Department of the University of Michigan, a rival of the Rush Medical College at Chicago, between which two schools a war of words has been waging for some time.

The apparently invidious remarks called forth by this enterprise of ours reminds us of an anecdote of a certain lady, a great belle, who had three rival suitors, each pressing his claims with great earnestness. One day the three met in the parlor of the lady's residence, when, seeing dark clouds gathering on their brows, and trouble brewing, she said to them — "Bide your time, gentlemen; *I'll have you all before I die*"—and—so she did! Gentlemen, please make the application.

We are under obligations to our friend, the editor of the *Buffalo Journal*, for his attempted explanation of our course, but would correct a misapprehension he labors under, in attributing the authorship of the biographies of the Presidents of the American Medical Associations to Dr. Davis. He had nothing to do with any of them, beyond furnishing the data for the biography of his own life, and but one of them, that of Dr. Chapman, was written by the editor of this journal.—*N. Y. Med. Reporter.*

